# Heterocyst Thylakoid Bioenergetics

**DOI:** 10.3390/life9010013

**Published:** 2019-01-25

**Authors:** Ann Magnuson

**Affiliations:** Department of Chemistry—Ångström, Uppsala University, Box 523, 75120 Uppsala, Sweden; ann.magnuson@kemi.uu.se; Tel.: +46-(0)18-471-6582

**Keywords:** cyanobacteria, thylakoid, photosynthesis nitrogen fixation, ferredoxin, biofuel

## Abstract

Heterocysts are specialized cells that differentiate in the filaments of heterocystous cyanobacteria. Their role is to maintain a microoxic environment for the nitrogenase enzyme during diazotrophic growth. The lack of photosynthetic water oxidation in the heterocyst puts special constraints on the energetics for nitrogen fixation, and the electron transport pathways of heterocyst thylakoids are slightly different from those in vegetative cells. During recent years, there has been a growing interest in utilizing heterocysts as cell factories for the production of fuels and other chemical commodities. Optimization of these production systems requires some consideration of the bioenergetics behind nitrogen fixation. In this overview, we emphasize the role of photosynthetic electron transport in providing ATP and reductants to the nitrogenase enzyme, and provide some examples where heterocysts have been used as production facilities.

## 1. Introduction

Oxygenic photosynthesis revolutionized life on earth by providing an endless source of energy and electrons for carbon dioxide fixation and by changing the composition of the atmosphere that enabled the development of multicellular life forms. Thylakoid membranes, the membrane system of oxygenic photoautotrophs, convert solar energy to biochemical energy via an electrochemical potential that drives ATP synthesis. The enzyme composition of thylakoid membranes is for the most part very similar in cyanobacteria, algae, and higher plants. Algae and higher plants have separate cellular compartments for photosynthesis and respiratory energy conversion. In cyanobacteria, the thylakoid membrane, where oxygen is produced, and the cell membrane, where most of the respiratory enzymes are located, are discrete zones of the same continuous membrane system [1]. Some of the respiratory enzymes are equally distributed between the two membrane domains in cyanobacteria, making the bioenergetics of these organisms complex and fascinating.

Several cyanobacterial strains are able to fix atmospheric nitrogen into ammonia. This is made by the enzyme nitrogenase, in an extremely ATP-demanding reaction:

N_2_ + 8 H^+^ + 8 e^−^ + 16 MgATP → 2 NH_3_ + H_2_ + 16 MgADP + 16 P_i_. (1)

To protect the nitrogenase from being inactivated by oxygen, nitrogen fixation has to be kept separated from photosynthetic oxygen formation either spatially or temporally. Some filamentous strains keep the nitrogenase away from the oxygen rich surroundings by differentiating a fraction of the cells into so-called heterocysts, where nitrogen fixation can take place in a safely microoxic environment [2,3]. In the heterocysts, Photosystem II (PSII) is necessarily inactivated, so the electron source for nitrogen fixation is provided by vegetative cells in the filament. Carbohydrates originating from photosynthetic carbon fixation are transported from the vegetative cells into the heterocysts where they are metabolized by the enzymes in the oxidative pentose phosphate cycle, generating NADPH [4,5,6].

Exactly which pathway the reducing equivalents take from there before they are fed to the nitrogenase by ferredoxin is still a matter of debate [7]. This question has gained in importance during the past decade, as heterocysts have become attractive candidates for being used as host compartments for oxygen-sensitive biosynthetic production [8,9]. To engineer non-native energy-consuming processes so that they are energy-efficient and non-harmful to the host, it is helpful to understand the bioenergetics of heterocysts and how reductants are utilized. The aim of this review is to shed some light on energy flows in the heterocyst and to indicate new research directions.

## 2. Enzymes in the Heterocyst Thylakoids

Heterocysts differentiate from the mature vegetative cells that are actively photosynthesizing in filamentous cyanobacteria [10]. The metabolism of the heterocyst is very different from the vegetative cells. During differentiation, a large number of genes are divergently regulated, and a thick cell wall is created to reduce the gas diffusion rate across the cell boundaries [11,12,13]. For reasons that are not well understood, the thylakoid membranes themselves also undergo morphological changes that seem to involve some re-distribution of proteins [14]. The enzyme composition of the thylakoid membrane in the heterocyst is not very different from that in vegetative cells, regarding which enzymes are there, but the amounts and relative stoichiometries of these enzymes change during heterocyst differentiation [15,16]. An additional membrane domain develops close to the polar nodules during heterocyst differentiation, dubbed “honeycombs” due to their appearance. These are rich in terminal oxidases which contribute to maintaining the microoxic environment. Because of this functional distinction from the thylakoid membranes, the polar honeycombs are outside the scope of this review (but see [14,17,18]). Several proteomic and spectroscopic studies have been done in order to understand the role of the thylakoid membrane in heterocyst bioenergetics, but detailed information on the dynamics and interrelationships of the energy-converting enzymes is still lacking.

### 2.1. Photosystem II

The oxygen-sensitivity of the nitrogenase demands that oxygen is actively removed from the heterocysts, explaining their high dependency on respiration. The removal of either terminal oxidases or heterocyst-specific flavodiiron proteins will cause the impairment of nitrogen fixation, as these are involved in oxygen scavenging [17,19,20]. Since oxygen evolution from PSII cannot be tolerated in heterocysts, it is often erroneously assumed that heterocysts lack PSII altogether. This misconception stems partly from early investigations, where harsh isolation methods unintentionally damaged the photosynthetic enzymes more than the nitrogenase, and resulted in the misunderstanding that heterocysts are brownish rather than green in color and completely lacking in chlorophyll-*a* fluorescence emission [21]. More recently, single cell spectroscopy has shown that heterocysts emit chlorophyll-*a* (Chl-*a*) fluorescence to lower but still comparable levels, as in vegetative cells [18,22]. Intact PSII complexes have been found in heterocysts from the cyanobacterium *Nostoc punctiforme* (PCC 73102/ATCC 29133, henceforth referred to as *N. punctiforme*). The purified heterocyst thylakoids were active in electron transport from the artificial electron donor DPC (1,5-diphenylcarbazide) to DCPIP (2,6-dichlorophenol indophenol) [15]. This activity is known to be specific for PSII-based electron transfer, but no water oxidation could be detected.

To preserve a microoxic environment then, PSII is seemingly only inactivated, not absent, in the heterocyst. Absorption and fluorescence spectral imaging performed on heterocysts in vivo showed that Chl-*a* fluorescence in heterocysts is dominated by Photosystem-I (PSI), but PSII-based fluorescence could still be observed [22]. In addition, the protein composition of PSII is to a large extent the same as in vegetative cells. Interestingly, in a large-scale proteomic analysis only the PsbW subunit was found in significantly lower amounts in heterocysts [16]. This protein has been shown to play a role in the formation and stabilization of the supramolecular organization of PSII in higher plants [23]. Exactly how inactivation of PSII is accomplished in heterocysts is not understood, but the loss of PsbW indicates that the mechanism for inactivation involves disruption of the supramolecular organization. Although the abundance of PSII in heterocysts is less than in vegetative cells, results suggest that the complexes are largely intact and could be re-activated.

### 2.2. Photosystem I

The nitrogenase reaction requires large amounts of ATP: for each N_2_ molecule that is reduced, the enzyme needs 16 ATP molecules to be hydrolyzed. The ATP requirement makes biological nitrogen fixation very nearly as energy-demanding as the industrial Haber–Bosch process [24]. The driving force for ATP synthesis is provided by PSI, which is highly active in the heterocyst, leading to a significantly higher nitrogenase activity under illumination than in the dark [25,26,27]. PSI drives light-induced electron transport from plastocyanin or cytochrome-c on the lumenal side, via ferredoxin to NADP+ on the cytoplasmic (stromal) side of the thylakoid membrane. In addition to this one-way electron donation to NADP+, PSI also performs so-called cyclic electron transport, where reducing equivalents from PSI are transferred to the plastoquinone pool, via the NADH dehydrogenase-like enzyme NDH-1. The reduced form of plastoquinone (plastoquinol) is re-oxidized by the cytochrome-*b*_6_/*f* (Cyt-*b*_6_/*f*) complex, and electrons are carried from Cyt-*b*_6_/*f* back to PSI by plastocyanin, which closes the circle.

Although some ATP synthesis can take place in the dark, via respiration or the glycolysis pathway, it has been established that the main ATP production in the heterocyst is driven by light reactions in the heterocyst thylakoids [25,28,29]. PSI consists of a heterodimer of the homologous transmembrane subunits PsaA and PsaB, as well as several accessory subunits. The necessity of PSI in heterocysts is underscored by the large increase in the abundance of PSI protein subunits during heterocyst differentiation and in the mature heterocysts [16,30]. Cyclic electron transport (CET), which fuels the proton gradient without generating NADPH, is an important role for PSI in all cyanobacteria and in plant chloroplasts, where it is essential for the survival of the plant [31,32]. It has been argued that CET is the only PSI-based electron transport in the heterocyst, and the only mechanism of ATP generation. However, linear electron transport is possible in the heterocyst. After carbohydrates are transported from the vegetative cells into the heterocysts, they are metabolized in the oxidative pentose pathway to generate NADPH, which supplies enough reducing equivalents for nitrogenase to reduce N_2_ all the way to NH_3_ (Equation (1)) [33,34]. It is possible, but not evident, that electrons are fed through the thylakoid membrane via the proton-pumping NDH-1 complex, thus generating some ATP on their way to nitrogenase (see below). This possible electron transport route is different from how the bioenergetic mechanisms of heterocysts are generally described but offers some interesting engineering options that are discussed later.

### 2.3. Cytochrome-b_6_/f

Cyanobacterial nitrogenase activity is stimulated by light. Early on, there was some ambiguity as to whether or not the immediate stimulation of nitrogenase by light was due to light reactions inside the heterocysts, or the photosynthetic activity in the vegetative cells. When it was shown that nitrogenase activity in isolated heterocysts can be interrupted by addition of dibromothymoquinone (DBMIB), an inhibitor of the cytochrome-*b*_6_/*f* (Cyt-*b*_6_*f*) complex, it became clear that it is the heterocyst thylakoid membranes that provide the driving force for ATP-synthesis [7,35]. The occurrence of the Cyt-*b*_6_*f* complex in the biosphere is limited to cyanobacteria, green algae, and plants. Its structure and function is similar to the respiratory *bc*_1_-complex in mitochondria and other bacteria, but has some structural features and cofactors that are not found in *bc*_1_-complexes [36].

The Cyt-*b*_6_*f* complex oxidizes plastoquinone and contributes to the build-up of a transmembrane proton gradient. The pool of plastoquinone molecules in the membrane is reduced by PSII during linear electron transport, but is also recycled during CET. Which enzyme complexes that partake in CET varies significantly between the kingdoms, with major differences between higher plants, green algae, and cyanobacteria, and the details remain obscure. In green algae, it has been established that CET can proceed from PSI, via ferredoxin directly to the Cyt-*b*_6_*f* complex, and from there carried back to PSI. Electron transport from ferredoxin to Cyt-*b*_6_*f* has not been established in plant thylakoids, however. In cyanobacteria, the NDH-1 complex seems to play an important role for CET, but the details of how the NDH-1 and/or Cyt-*b*_6_*f* complexes are communicating with PSI during CET are still debated [37].

In heterocysts, the situation is even more unclear. Nitrogenase activity measurements in isolated heterocysts showed that the Cyt-*b*_6_*f* complex is essential for nitrogen fixation [35]. The details of which route the reductants take are still not known, however, and it has never been experimentally established that CET is the only light-driven mechanism for ATP production (see discussion in Section 3). In heterocyst-specific proteomic studies, several protein subunits of the Cyt-*b*_6_*f* complex were found in higher amounts in heterocysts than in vegetative cells, similar to the situation of PSI proteins that were also increased [15,16]. What can be concluded is that the Cyt-*b*_6_*f* complex is essential for ATP production and thereby for nitrogen fixation.

### 2.4. Ferredoxins

Ferredoxins are small, soluble electron transport proteins that are not permanently part of any membrane protein complex, but their central role in photosynthetic electron transfer grants them a special place in this overview. Ferredoxins, characterized by their redox-active iron-sulfur (FeS) cluster centers, mediate electron transfer in a range of metabolic reactions. Cyanobacteria possess a varying number of ferredoxins depending on the species. The primary electron acceptor from PSI in all oxygenic photosynthetic organisms is the so-called “plant-type” ferredoxin, often referred to as Fd-I, which contains a [2Fe-2S] cluster that can be reduced in a one-electron reaction from a formal charge of −2 to the −3 state. Under conditions of linear electron transfer, Fd-I carries one electron at a time from PSI to the ferredoxin-NADP-reductase (FNR), which reduces NADP to NADPH in a two-electron reaction.

The heterocystous cyanobacteria have evolved a second plant-type ferredoxin, FdxH, which is exclusively expressed in the heterocyst. Although the two ferredoxins have a modest 53% sequence identity, the overall protein structure and the [FeS]-cluster of FdxH is very similar to that of Fd-I. The major difference between FdxH and Fd-I is the redox potential, which is approximately 50 mV more positive in FdxH than in Fd-I [38,39]. Calculations of protein surface charges have suggested that FdxH, but not Fd-I, can associate with nitrogenase in a way that is constructive for electron transfer. Fd-I appears to be insufficient in promoting nitrogen fixation in vitro, in spite of its more negative midpoint potential, yet an *fdxH* deletion strain of *Nostoc*/*Anabaena* sp. PCC 7120 was still able to grow diazotrophically [40,41,42].

In all oxygenic photosynthetic organisms, Fd-I associates with FNR to reduce NADP+ to NADPH. In heterocysts, on the other hand, NADPH is formed by the oxidative pentose phosphate pathway. It has been proposed that NADPH reduces Fd via FNR directly, without involvement of PSI or the thylakoid electron transport chain. This is a thermodynamically unfavorable reaction except when the NADPH/NADP+ ratio is very high, as it is likely to be in the heterocysts [43,44]. Still, this explanation cannot account for the fact that nitrogenase activity can be inhibited by DBMIB, which blocks electron transport from the Cyt-*b*_6_*f* complex [35].

Several cyanobacterial strains are known to express two different isoforms of FNR. The two isoforms differ in size and are both transcribed from the same gene, *petH*. In *Nostoc/Anabaena* sp. PCC 7120 (henceforth referred to as *Nostoc* PCC 7120), *petH* has two alternative transcriptional start points that determine the length of the translated amino acid chain [45,46,47]. Transcripts for the small isoform have been found in nitrogen fixing filaments and in heterocysts [48]. In a comparative study between the non-diazotrophic cyanobacterium *Synechocystis* PCC6803 and the nitrogen-fixing strain *Anabaena variabilis*, the two isoforms were quantified under different growth conditions. It was concluded that the small isoform accumulates in nitrogen-fixing cultures and is predominantly found in the soluble cell fraction, whereas the larger isoform is transcribed under normal, photosynthetic conditions and is localized to the membrane fraction [49]. It has therefore been concluded that the soluble form of FNR could provide a shortcut for electron transfer from NADPH to nitrogenase via FdxH, while the membrane-bound form of FNR would be too fixed at the membrane surface to make electron transfer to nitrogenase efficient enough. On the other hand, the association between ferredoxin and FNR is not permanent, and unless there are other unknown protein factors keeping them together there is nothing to prevent FdxH (or Fd-I if it is present) from donating electrons elsewhere. There are also other possibilities for electron donation from FdxH that should be considered, which will now be discussed.

### 2.5. The Type-1 NADH Dehydrogenase

The existence of the NADH dehydrogenase (NDH-1) was discovered as a part of the cyanobacterial thylakoid membrane in the mid 1980s, two decades after the photosystems [50,51]. The NDH-1 is an adaptive enzyme that can have a slightly different composition depending on metabolic requirements. Blue native gel electrophoretic separation of thylakoid protein complexes, in combination with mass spectrometry, has enabled the characterization of different variants of NDH-1 in cyanobacteria (reviewed in [37]). Some variants are thought to be prevalent in the thylakoid membranes close to the photosynthetic enzymes and to be involved in CO_2_ concentration. Other subunit compositions of NDH-1 are more common in membrane regions where respiration dominates. The structure of the cyanobacterial NDH-1 has been modeled on the respiratory Complex-1, based on results from membrane proteomics and comparisons between the crystal structure of Complex I and the single-particle imaging of cyanobacterial NDH-1 [52,53].

The NDH-1 complex is involved in CET, where reducing equivalents are transferred from PSI back to the quinone pool in the thylakoid membrane [37]. In plant thylakoid membranes, the NDH-1 has been known to form supercomplexes with PSI, possibly enhancing the CET efficiency. Anything similar has so far not been observed in cyanobacteria, but the NdhP subunit of the NDH-1 complex can improve CET efficiency by sustaining a coupling with PSI [54].

Many efforts have been made to discover the identity of the electron-donating partner to NDH-1. In mitochondria, the electron donor to Complex I is NADH or NADPH, and the electron acceptor is ubiquinone. The cyanobacterial NDH-1 has been found to perform NADPH-dependent electron transfer to plastoquinone, and others and we have argued that NADPH would be the electron donor to NDH-1 in cyanobacteria [7,55,56]. On the other hand, homologues to the NADH-binding domain in mitochondrial Complex I are missing from cyanobacterial NDH-1, and recent studies indicate that the direct electron donor to NDH-1 is ferredoxin [37]. In chloroplasts of *Arabidopsis thaliana*, NADPH-dependent plastoquinone reduction was shown to be dependent on the presence of ferredoxin, and a protein, CRR31, was found to be necessary for the high-affinity binding of ferredoxin to plastid NDH-1 [57,58]. In cyanobacteria, a small protein that is homologous to the C-terminal of CRR31, denoted NdhS, has been found in the hydrophilic, n-side, domain of NDH-1 complexes. It seems to be conserved across the phylum and has been shown to interact with Fd-I in vitro [59,60].

In heterocysts, several of the soluble and membrane-bound protein subunits from NDH-1 have been detected [15,16]. As with PSI and Cyt-*b*_6_*f*, the NDH-1 proteins were found in a higher abundancy in heterocysts than in vegetative cells. Together with the other proteins that are upregulated in heterocysts, this indicates that NDH-1 is needed to support CET in heterocysts, as in vegetative cells. However, because the cyanobacterial NDH-1 complex is exposed not just to the photosynthetic proteins but also to the entire cytoplasm, it may also play a role in directing reductants from other metabolic pathways to the thylakoid membrane. Despite its main role in the heterocyst, it could also participate in linear electron transfer from ferredoxin to nitrogenase. Interestingly, the NdhS subunit was identified from *N. punctiforme* (Npun_R5164) in a heterocyst-specific proteomic study, and was also similarly upregulated in comparison to the other NDH-1 proteins [16]. This leads to the interesting inference that not only is NDH-1 essential for heterocyst function, but it is most likely dependent on ferredoxin as its electron donor in the heterocyst as well.

## 3. Balancing the Electron/Proton Budget

How can we reconcile the need for ferredoxin to support CET, with the need for efficient electron transfer from NADPH to nitrogenase, if ferredoxin is the primary electron donor to both NDH-1 and nitrogenase? The high consumption of energy and reductants by nitrogenase would seem to demand tightly controlled routes of electron transport. It is easy to picture the heterocyst-specific FdxH as feeding electrons exclusively to nitrogenase, while the photosynthetic Fd-I is participating in CET around PSI. Experimental evidence for this is lacking, however, and proteomic analyses have failed to quantify the amount of Fd-I and FdxH in heterocysts [16,30]. The redox potential of the iron protein (NifH), which is the part of nitrogenase that accepts electrons from ferredoxin, has not been determined in cyanobacteria. Even in *Azotobacter vinelandii*, a better-studied model strain for nitrogen fixation, difficulties of determining the physiologically relevant reduction potential are prevailing. Measurements in vitro have indicated that the Fe-S cluster of NifH in the “resting” state has a midpoint potential of approximately −310 mV, and that this shifts by at least 100 mV to the negative upon nucleotide binding [61,62]. If the NifH in heterocystous cyanobacteria has a similar reduction potential, it is possible for FdxH to act as a reductant within its physiologically relevant potential [38,39].

As already described, nitrogenase is not the only available electron acceptor. The NDH-1 complex offers itself an equally favorable electron-accepting partner. Which of the two final acceptors “wins” the electron might depend on the reduction level, the physical proximity, or both. Since the nitrogenase reaction is rather slow, the NDH-1 complex may provide a suitable passage for some of the reducing equivalents arriving at its threshold. The possibility of adding an electron flux to the proton translocating processes may prove this flexibility in electron transport pathways to be an advantage.

As noted above, the ATP which is provided to nitrogenase is almost exclusively synthesized by light-driven processes in the heterocyst. Aproton gradient across the heterocyst thylakoid membrane is built up via light reactions and provides the driving force for the membrane-bound ATP-synthase [28,29]. The energy requirement for ATP synthesis depends on the magnitude of the gradient, the concentrations of ATP and ADP, and on the H+/ATP ratio of the ATP synthase [63]. In addition, the H+/ATP ratio depends on the size of the c-ring in the membrane domain (F_0_) of ATP synthase, where protons from the lumenal side of the membrane are translocated to the stromal/cytoplasmic side. The subunit composition of the c-ring has been investigated in several cyanobacterial strains, including the heterocystous strain *Nostoc* PCC7120 and was found to contain on average of 14 copies of the c-subunit in most cyanobacteria [64]. This implicates that 4–5 H+ need to be translocated across the membrane per ATP molecule that is synthesized. This is a lower ATP/H+ ratio than in the mammalian mitochondrial ATP-synthase, but similar to that in plant thylakoids. ATP synthesis in thylakoid membranes is thus more weakly coupled to electron transfer than in mammalian respiratory chains, meaning that more electrons have to be transported through the thylakoid membrane to generate a sufficient proton gradient for ATP synthesis to occur.

By analogy with what is known about the mitochondrial Complex I, every two electrons that are donated by ferredoxin to NDH-1 in the thylakoid membrane should result in the translocation of 4 H+ to the lumenal side. The Cyt-*b*_6_*f* complex has the same e-/H+ stoichiometry. This means that one ATP molecule—at most—can be obtained when one electron is donated by ferredoxin and transported through the thylakoid membrane. CET, on the other hand, may produce enough ATP independently of electron transport from NADPH. Thus, transporting all electrons from ferredoxin via the thylakoid membrane may be an unfavorable detour, considering that the nitrogenase reaction demands two ATP molecules for every reduction. The alternative is then that electrons are donated directly from the FNR/Fd reaction to the nitrogenase without passing the thylakoid membrane, as was suggested above.

**Figure 1:**
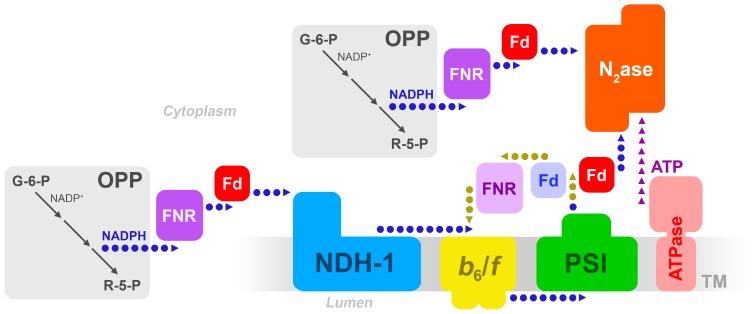
Possible electron transfer pathways in the heterocyst, from carbohydrate breakdown to nitrogenase, involving the thylakoid membrane. Adapted from [7]. OPP = oxidative pentose phosphate pathway; FNR = ferredoxin/NADP+ reductase; Fd = ferredoxin; *b*_6_/*f* = the cytochrome-*b*_6_/*f* complex; PSI = Photosystem I; N_2_ase = nitrogenase. The blue (light) Fd denotes Fd-I, and the red (dark) Fd is the heterocyst-specific FxdH. The light-colored FNR denotes the long form of FNR, the darker-colored denotes the truncated, or short, form of FNR.

The classical picture where ATP is generated purely via CET, independent of electron transfer to nitrogenase, is appealingly simple. Nevertheless, the nitrogenase reduction reaction is painstakingly slow, and the fate of a reduced ferredoxin will inevitably be decided by redox equilibria as well as by kinetics. If all NifH proteins have been reduced, a pool of reduced FdxH may turn to deliver electrons to NDH-1 (Figure 1). What is missing is a detailed picture of the supramolecular organization of enzymes inside the heterocysts. We know almost nothing of where or how enzymes are located relative to each other, and a full understanding of heterocyst bioenergetics will be lacking until this can be revealed.

## 4. Heterocysts as Cell Factories

### 4.1. Biotechnological Uses of Heterocysts

The interest in heterocystous cyanobacteria has increased during the past decade. The oxygen-free environment offered by the heterocysts, in combination with an increasing arsenal of molecular biology tools for heterocyst-specific expression, has made the exploitation of heterocysts as cell factories an interesting option [65,66]. With photosynthesis as a base energy provider, the option for energy-lean and additive-free production becomes an attractive possibility, in particular for energy applications [67]. Biofuel production from genetically engineered cyanobacteria is a promising route towards a carbon-neutral fuel production and independence from fossil fuels. What needs to be considered is the carbon source, which needs to be imported from vegetative cells, and whether economizing the ATP and reductants available in heterocysts can be improved.

#### 4.1.1. Alcohol Production

Cyanobacteria have been engineered for the production of chemicals that can be used directly as fuels, such as ethanol and butanol, albeit at low production efficiencies [68,69]. Production of ethanol via fermentation generally occurs under low oxygen pressure, and it can therefore be challenging to combine ethanol production with oxygenic photosynthesis, or at least with full photoautotrophic growth. Fermentative metabolism is not the normal growth mode for the unicellular cyanobacterium *Synechocystis* PCC 6803, but it is possible to grow it under photoheterotrophic, or completely heterotrophic conditions in darkness, using glucose as a carbon source [70,71]. *Synechocystis* PCC 6803 is known to possess an endogenous enzyme resembling alcohol dehydrogenase, AdhA, which is essential for heterotrophic growth and which can be activated towards ethanol production [72,73].

A way to increase ethanol production under photoautotropic conditions is to clone existing ethanol production pathways into the heterocysts of filamentous cyanobacteria. In a recent study, pyruvate decarboxylase of *Zymomonas mobilis* and the alcohol dehydrogenase *adhA* from *Synechocystis* sp. PCC 6803 were expressed in heterocysts of *Nostoc* PCC 7120. An unusually high ethanol production was then observed simultaneously with nitrogen fixation [74]. Interestingly, when the utilization of sucrose in the heterocysts was increased by expressing the invertase *invB* under a heterocyst-specific promoter, the *Anabaena* strain expressing *invB* showed the highest ethanol production that could be obtained, all other things being equal. Although this increased the availability of reductants in the heterocysts, the ethanol production was still limited by the photosynthetic production in the vegetative cells. The available CO_2_ was therefore increased from atmospheric concentration to 1%, and evaporated ethanol was at the same time stripped from the cultures to avoid ethanol poisoning. This led to a seven-fold increase in the ethanol production, and the productivity continued for a longer time. The authors did not show which of the two changes had the greatest effect on the production, or if the productivity could be further enhanced by higher CO_2_ concentrations.

#### 4.1.2. H_2_ Production

Hydrogen gas (H_2_) is a fuel that is projected to become an important alternative to fossil fuels. When H_2_ is burned, or used in a fuel cell, the only waste product is water vapor, so a major effort to use H_2_ as energy carrier can have a positive environmental impact. One possibility for sustainable production of H_2_ is offered by photosynthetic microorganisms, and research in cyanobacterial H_2_ production has increased rapidly since the beginning of the 21st century. Most hydrogenases are very sensitive to inactivation by oxygen, however, and H_2_ production by unicellular cyanobacteria can therefore only be accomplished in the dark or by strict control of the photosynthetic apparatus. Heterocystous cyanobacteria produce some H_2_ naturally by the nitrogenase reaction, and several attempts to increase nitrogenase-based production have been made [27,75].

H_2_-production from nitrogenase has poor energy efficiency, and efforts to increase it, such as manipulating the metal content or excluding N_2_ from the atmosphere, will inevitably be detrimental to nitrogen fixation and growth. Heterologous expression of hydrogenases in heterocysts is therefore an attractive alternative. This has been applied by the heterologous expression of [FeFe] hydrogenases from, e.g., the facultative anaerobe *Shewanella oneidensis* and from *Clostridium acetobutylicum* in the heterocysts of *Nostoc* PCC 7120 [9,76]. Due to the considerable oxygen sensitivity of HydA from *C. acetobutylicum*, in vivo activity from the engineered strain could only be observed by inhibiting PSII activity with DCMU (3-(3,4-dichlorophenyl)-1,1-dimethylurea) or by expressing the oxygen scavenger cyanoglobin in the heterocysts [9]. H_2_ evolution could then be sustained for 100 h, which is good but somewhat puzzling. Nitrogenase activity measured in several heterocystous cyanobacteria during diurnal light–dark cultivation, or during inhibition of PSII by DCMU, showed that acetylene reduction by nitrogenase could be sustained for about 6 h after the onset of “night” or the inhibition of PSII, respectively [33,77]. Although the specific time span for the depletion of intracellular carbon supplies might be prolonged, the question beckons to be answered of how reductants could be available to HydA in heterocysts for the duration of the experiment by Avilan et al. [9].

In a different study, a modified variant of the uptake hydrogenase HupSL, a NiFe enzyme, was homologously expressed in heterocysts of a HupSL^-^ variant of its native host strain *N. punctiforme* [78]. A point mutation in the hydrogenase small subunit, HupS, was shown by EPR spectroscopy to result in a conversion of one of the FeS clusters in HupS, from a [4Fe-4S] cluster into a [3Fe-4S] cluster. The modified hydrogenase was introduced on an expression vector, under a synthetic heterocyst-specific promoter. In the beginning of the cultivation period after removal of nitrate, the in vivo H_2_ evolution was indistinguishable from nitrogenase-based H_2_ evolution. After four days, however, when the activity and energy consumption by nitrogenase started to decline, a sustained H_2_ production was observed in the strain carrying the modified hydrogenase. The result demonstrated that, as long as reductants are not totally consumed by nitrogenase, prolonged H_2_ production from an engineered hydrogenase can be supported in a photoautotrophically growing cyanobacterium.

### 4.2. Squeezing the Electron Budget

A major limitation with using heterocysts as cell factories lies in the limited availability of reductants and/or carbon sources. This may be alleviated by increasing the external CO_2_ pressure, which increases the carbon supply for photosynthesis in the vegetative cells, or by enhancing sugar breakdown in the heterocysts [72,74]. A more sophisticated method is to introduce synthetic metabolic pathways to increase the rate of CO_2_ fixation by the Calvin–Benson–Bassham cycle, which was recently and elegantly shown to enhance ethanol production in *Synechocystis* PCC 6803 [79].

If reductants are the limiting factor, one way of making the most of a tight electron budget in the heterocysts could be to restrict electron transport to nitrogenase. The heterocyst-specific ferredoxin FdxH (Section 2.4) is important for efficient electron donation to nitrogenase, but an FdxH deletion mutant was still able to grow diazotrophically at a reduced rate [42]. With a lower electron donation rate to nitrogenase, other production pathways could be favored. This was shown in the study where a modified uptake hydrogenase was expressed in heterocysts of *N. punctiforme* and produced H_2_ after the nitrogenase activity had started to decline [78].

There may be a different strategy, which so far is mainly on the drawing table. It involves the ring stoichiometry of the ATP synthase. The proton gradient across the thylakoid membrane is coupled mechanically to ATP synthesis by the rotation of the ring of c-subunits in the membrane domain (F_0_) of ATP synthase. Each c-subunit translocates one proton from the lumenal side during a full 360° rotation of the ring, leading to the synthesis of three ATP molecules. The proton requirement for ATP synthesis thus depends on the number of c-subunits [80]. The subunit composition of the c-ring varies largely between the kingdoms, and the smallest rings consisting of eight c-subunits have so far only been found in mammals. The H+/ATP ratio for mammalian ATP synthase is 2.7, whereas in bacterial enzymes where the ring can contain up to 15 c-subunits, the ratio is as high as 5. If the H+/ATP ratio could be lowered, it would mean a better electron “economy” since fewer reducing equivalents would be needed for generating the transmembrane proton gradient.

A recent investigation of c-subunit structure and inter-subunit interactions in the ATP synthase of *Ilyobacter tartaricus* revealed that a conserved motif of glycines (GxGxGxGxG) promote dimerization and had a direct effect on the c-subunit stoichiometry [64]. By introducing mutations in this motif, it was possible to alter the stoichiometry from c_11_ to c_12_ and concomitantly increase the H+/ATP ratio from 3.7 to 4. It is too early to say if it would be possible to alter a bacterial c-ring to resemble the mammalian assembly or if it would be possible to drive ATP synthesis in a bacterium with it.

## 5. Conclusions

The bioenergetics of the thylakoid membranes in heterocysts is still far from being as well known or investigated as that of the “normal” photosynthesizing cyanobacterial cells. The growing interest for heterocysts as cell factories evokes questions about energy utilization by heterocystous cyanobacteria, and about how it can be made more efficient for biotechnological applications. The aim of this review was to cast light over different aspects of heterocyst bioenergetics and to provide some starting points for further engineering endeavors.

## References

[1] Mullineaux C.W. (2014). Co-existence of photosynthetic and respiratory activities in cyanobacterial thylakoid membranes. Biochim. Biophys. Acta.

[2] Fay P. (1992). Oxygen Relations of Nitrogen-Fixation in Cyanobacteria. Microbiol. Rev..

[3] Gallon J.R. (1992). Reconciling the incompatible N_2_ fixation and O_2_. New Phytol..

[4] Cumino A.C., Marcozzi C., Barreiro R., Salerno G.L. (2007). Carbon cycling in Anabaena sp PCC 7120. Sucrose synthesis in the heterocysts and possible role in nitrogen fixation. Plant Physiol..

[5] Curatti L., Giarrocco L.E., Cumino A.C., Salerno G.L. (2008). Sucrose synthase is involved in the conversion of sucrose to polysaccharides in filamentous nitrogen-fixing cyanobacteria. Planta.

[6] Nurnberg D.J., Mariscal V., Bornikoel J., Nieves-Morion M., Krauss N., Herrero A., Maldener I., Flores E., Mullineaux C.W. (2015). Intercellular diffusion of a fluorescent sucrose analog via the septal junctions in a filamentous cyanobacterium. mBio.

[7] Magnuson A., Cardona T. (2016). Thylakoid membrane function in heterocysts. BBA-Bioenergetics.

[8] Liu H.Y., Ni J., Xu P., Tao F. (2018). Enhancing Light-Driven 1,3-Propanediol Production by Using Natural Compartmentalization of Differentiated Cells. ACS Synth. Biol..

[9] Avilan L., Roumezi B., Risoul V., Bernard C.S., Kpebe A., Belhadjhassine M., Rousset M., Brugna M., Latifi A. (2018). Phototrophic hydrogen production from a clostridial FeFe hydrogenase expressed in the heterocysts of the cyanobacterium Nostoc PCC 7120. Appl. Microbiol. Biotechnol..

[10] Meeks J.C., Campbell E.L., Summers M.L., Wong F.C. (2002). Cellular differentiation in the cyanobacterium Nostoc punctiforme. Arch. Microbiol..

[11] Golden J.W., Yoon H.S. (2003). Heterocyst development in Anabaena. Curr. Opin. Microbiol..

[12] Flores E., Herrero A. (2010). Compartmentalized function through cell differentiation in filamentous cyanobacteria. Nat. Rev. Microbiol..

[13] Herrero A., Picossi S., Flores E., Chauvat F., Cassier-Chauvat C. (2013). Gene expression during heterocyst differentiation. Advances in Botanical Research, Genomics of Cyanobacteria.

[14] Santamaría-Gómez J., Mariscal V., Luque I. (2018). Mechanisms for Protein Redistribution in Thylakoids of Anabaena during Cell Differentiation. Plant Cell Physiol..

[15] Cardona T., Battchikova N., Zhang P.P., Stensjo K., Aro E.M., Lindblad P., Magnuson A. (2009). Electron transfer protein complexes in the thylakoid membranes of heterocysts from the cyanobacterium *Nostoc punctiforme*. Biochim. Biophys. Acta.

[16] Ow S.Y., Noirel J., Cardona T., Taton A., Lindblad P., Stensjo K., Wright P.C. (2009). Quantitative overview of N_2_ fixation in *Nostoc punctiforme* ATCC 29133 through cellular enrichments and iTRAQ shotgun proteomics. J. Proteome Res..

[17] Valladares A., Maldener I., Muro-Pastor A.M., Flores E., Herrero A. (2007). Heterocyst development and diazotrophic metabolism in terminal respiratory oxidase mutants of the cyanobacterium Anabaena sp strain PCC 7120. J. Bacteriol..

[18] Kumazaki S., Akari M., Hasegawa M. (2013). Transformation of Thylakoid Membranes during Differentiation from Vegetative Cell into Heterocyst Visualized by Microscopic Spectral Imaging. Plant Physiol..

[19] Allahverdiyeva Y., Mustila H., Ermakova M., Bersanini L., Richaud P., Ajlani G., Battchikova N., Cournac L., Aro E.M. (2013). Flavodiiron proteins Flv1 and Flv3 enable cyanobacterial growth and photosynthesis under fluctuating light. Proc. Natl. Acad. Sci. USA.

[20] Allahverdiyeva Y., Isojarvi J., Zhang P., Aro E.M. (2015). Cyanobacterial oxygenic photosynthesis is protected by flavodiiron proteins. Life.

[21] Magnuson A., Cardona T. (2018). Isolation of Intact Thylakoid Membranes from Heterocysts of Filamentous, Nitrogen-Fixing Cyanobacteria. Methods Mol. Biol..

[22] Nozue S., Katayama M., Terazima M., Kumazaki S. (2017). Comparative study of thylakoid membranes in terminal heterocysts and vegetative cells from two cyanobacteria, Rivularia M-261 and Anabaena variabilis, by fluorescence and absorption spectral microscopy. BBA-Bioenergetics.

[23] Plochinger M., Schwenkert S., von Sydow L., Schroder W.P., Meurer J. (2016). Functional Update of the Auxiliary Proteins PsbW, PsbY, HCF136, PsbN, TerC and ALB3 in Maintenance and Assembly of PSII. Front. Plant Sci..

[24] Burgess B.K., Lowe D.J. (1996). Mechanism of molybdenum nitrogenase. Chem. Rev..

[25] Almon H., Bohme H. (1982). Photophosphorylation in isolated heterocysts from the blue-green-alga *Nostoc muscorum*. Biochim. Biophys. Acta.

[26] Janaki S., Wolk C.P. (1982). Synthesis of nitrogenase by isolated heterocysts. Biochim. Biophys. Acta.

[27] Lindberg P., Schutz K., Happe T., Lindblad P. (2002). A hydrogen-producing, hydrogenase-free mutant strain of Nostoc punctiforme ATCC 29133. Int. J. Hydrog. Energ..

[28] Telor E., Stewart W.D.P. (1976). Photosynthetic electron transport, ATP synthesis and nitrogenase activity in isolated heterocysts of *Anabaena cylindrica*. Biochim. Biophys. Acta.

[29] Ernst A., Bohme H., Boger P. (1983). Phosphorylation and nitrogenase activity in isolated heterocysts from *Anabaena variabilis* (ATCC-29413). Biochim. Biophys. Acta.

[30] Ow S.Y., Cardona T., Taton A., Magnuson A., Lindblad P., Stensjo K., Wright P.C. (2008). Quantitative shotgun proteomics of enriched heterocysts from Nostoc sp. PCC 7120 using 8-plex isobaric peptide tags. J. Proteome Res..

[31] Munekage Y., Hashimoto M., Miyake C., Tomizawa K., Endo T., Tasaka M., Shikanai T. (2004). Cyclic electron flow around Photosystem I is essential for photosynthesis. Nature.

[32] Johnson G.N. (2011). Physiology of PSI cyclic electron transport in higher plants. BBA-Bioenergetics.

[33] Bottomley P.J., Stewart W.D.P. (1977). ATP And Nitrogenase Activity In Nitrogen-Fixing Heterocystous Blue-Green-Algae. New Phytol..

[34] Summers M.L., Wallis J.G., Campbell E.L., Meeks J.C. (1995). Genetic evidence of a major role for glucose-6-phosphate-dehydrogenase in nitrogen-fixation and dark growth of the cyanobacterium *Nostoc* sp strain ATCC-29133. J. Bacteriol..

[35] Ernst A., Bohme H. (1984). Control of hydrogen-dependent nitrogenase activity by adenylates and electron flow in heterocysts of *Anabaena variabilis* (ATCC-29413). Biochim. Biophys. Acta.

[36] Kurisu G., Zhang H.M., Smith J.L., Cramer W.A. (2003). Structure of the cytochrome b(6)f complex of oxygenic photosynthesis: Tuning the cavity. Science.

[37] Peltier G., Aro E.M., Shikanai T., Merchant S.S. (2016). NDH-1 and NDH-2 Plastoquinone Reductases in Oxygenic Photosynthesis. Annual Review of Plant Biology, Volume 67.

[38] Bohme H., Schrautemeier B. (1987). Comparative characterization of ferredoxins from heterocysts and vegetative cells of *Anabaena variabilis*. Biochim. Biophys. Acta.

[39] Hurley J.K., Weber-Main A.M., Stankovich M.T., Benning M.M., Thoden J.B., Vanhooke J.L., Holden H.M., Chae Y.K., Xia B., Cheng H. (1997). Structure-function relationships in Anabaena ferredoxin: Correlations between X-ray crystal structures, reduction potentials, and rate constants of electron transfer to ferredoxin:NADP^+^ reductase for site-specific ferredoxin mutants. Biochemistry.

[40] Schmitz S., Schrautemeier B., Bohme H. (1993). Evidence from directed mutagenesis that positively charged amino-acids are necessary for interaction of nitrogenase with the 2Fe-2S heterocyst ferredoxin (FdxH) from the cyanobacterium Anabaena sp, PCC7120. Mol. Gen. Genet..

[41] Hurley J.K., Schmeits J.L., Genzor C., GomezMoreno C., Tollin G. (1996). Charge reversal mutations in a conserved acidic patch in Anabaena ferredoxin can attenuate or enhance electron transfer to ferredoxin:NADP(+) reductase by altering protein/protein orientation within the intermediate complex. Arch. Biochem. Biophys..

[42] Masepohl B., Scholisch K., Gorlitz K., Kutzki C., Bohme H. (1997). The heterocyst-specific fdxH gene product of the cyanobacterium Anabaena sp. PCC 7120 is important but not essential for nitrogen fixation. Mol. Gen. Genet..

[43] Schrautemeier B., Bohme H., Boger P. (1984). In vitro studies on pathways and regulation of electron-transport to nitrogenase with a cell-free-extract from heterocysts of *Anabaena variabilis*. Arch. Microbiol..

[44] Schrautemeier B., Bohme H., Boger P. (1985). Reconstitution of a light-dependent nitrogen-fixing and transhydrogenase system with heterocyst thylakoids. Biochim. Biophys. Acta.

[45] Valladares A., Muro-Pastor A.M., Fillat M.F., Herrero A., Flores E. (1999). Constitutive and nitrogen-regulated promoters of the *petH* gene encoding ferredoxin:NADP^+^ reductase in the heterocyst-forming cyanobacterium *Anabaena* sp. FEBS Lett..

[46] Omairi-Nasser A., de Gracia A.G., Ajlani G. (2011). A larger transcript is required for the synthesis of the smaller isoform of ferredoxin: NADP oxidoreductase. Mol. Microbiol..

[47] Omairi-Nasser A., Galmozzi C.V., Latifi A., Muro-Pastor M.I., Ajlani G. (2014). NtcA is responsible for accumulation of the small isoform of ferredoxin:NADP oxidoreductase. Microbiology.

[48] Razquin P., Fillat M.F., Schmitz S., Stricker O., Bohme H., Gomez-Moreno C., Peleato M.L. (1996). Expression of ferredoxin-NADP^+^ reductase in heterocysts from *Anabaena* sp. Biochem. J..

[49] Alcantara-Sanchez F., Leyva-Castillo L.E., Chagolla-Lopez A., de la Vara L.G., Gomez-Lojero C. (2017). Distribution of isoforms of ferredoxin-NADP(+) reductase (FNR) in cyanobacteria in two growth conditions. Int. J. Biochem. Cell Biol..

[50] Meng B.Y., Matsubayashi T., Wakasugi T., Shinozaki K., Sugiura M., Hirai A., Mikami T., Kishima Y., Kinoshita T. (1986). Ubiquity of the genes for components of a NADH dehydrogenase in higher-plant chloroplast genomes. Plant Sci..

[51] Ogawa T. (1991). A gene homologous to the subunit-2 gene of nadh dehydrogenase is essential to inorganic carbon transport of synechocystis PCC6803. Proc. Natl. Acad. Sci. USA.

[52] Arteni A.A., Zhang P.P., Battchikova N., Ogawa T., Aro E.M., Boekema E.J. (2006). Structural characterization of NDH-1 complexes of Thermosynechococcus elongatus by single particle electron microscopy. BBA-Bioenergetics.

[53] Baradaran R., Berrisford J.M., Minhas G.S., Sazanov L.A. (2013). Crystal structure of the entire respiratory complex I. Nature.

[54] Schwarz D., Schubert H., Georg J., Hess W.R., Hagemann M. (2013). The Gene sml0013 of Synechocystis Species Strain PCC 6803 Encodes for a Novel Subunit of the NAD(P)H Oxidoreductase or Complex I That Is Ubiquitously Distributed among Cyanobacteria. Plant Physiol..

[55] Ma W.M., Deng Y., Ogawa T., Mi H.L. (2006). Active NDH-1 complexes from the cyanobacterium Synechocystis sp strain PCC 6803. Plant Cell Physiol.

[56] Mi H.L., Endo T., Ogawa T., Asada K. (1995). Thylakoid membrane-bound, NADPH-specific pyridine-nucleotide dehydrogenase complex mediates cyclic electron transport in the cyanobacterium *Synechocystis* sp PCC-6803. Plant Cell Physiol..

[57] Peng L.W., Shikanai T. (2011). Supercomplex Formation with Photosystem I Is Required for the Stabilization of the Chloroplast NADH Dehydrogenase-Like Complex in Arabidopsis. Plant Physiol..

[58] Yamamoto H., Peng L.W., Fukao Y., Shikanai T. (2011). An Src Homology 3 Domain-Like Fold Protein Forms a Ferredoxin Binding Site for the Chloroplast NADH Dehydrogenase-Like Complex in Arabidopsis. Plant Cell.

[59] Battchikova N., Wei L.Z., Du L.Y., Bersanini L., Aro E.M., Ma W.M. (2011). Identification of Novel Ssl0352 Protein (NdhS), Essential for Efficient Operation of Cyclic Electron Transport around Photosystem I, in NADPH:plastoquinone Oxidoreductase (NDH-1) Complexes of Synechocystis sp. PCC 6803. J. Biol. Chem..

[60] He Z.H., Zheng F.F., Wu Y.Z., Li Q.H., Lv J., Fu P.C., Mi H.L. (2015). NDH-1L interacts with ferredoxin via the subunit NdhS in Thermosynechococcus elongatus. Photosynth. Res..

[61] Lanzilotta W.N., Ryle M.J., Seefeldt L.C. (1995). Nucleotide hydrolysis and protein conformational-changes in azotobacter-vinelandii nitrogenase iron protein—Defining the function of aspartate-129. Biochemistry.

[62] Jasniewski A.J., Sickerman N.S., Hu Y.L., Ribbe M.W. (2018). The Fe Protein: An Unsung Hero of Nitrogenase. Inorganics.

[63] Muller V., Hess V. (2017). The Minimum Biological Energy Quantum. Front. Microbiol..

[64] Pogoryelov D., Klyszejko A.L., Krasnoselska G.O., Heller E.M., Leone V., Langerd J.D., Vonck J., Muller D.J., Faraldo-Gomez J.D., Meier T. (2012). Engineering rotor ring stoichiometries in the ATP synthase. Proc. Natl. Acad. Sci. USA.

[65] Srirangan K., Pyne M.E., Chou C.P. (2011). Biochemical and genetic engineering strategies to enhance hydrogen production in photosynthetic algae and cyanobacteria. Bioresour. Technol..

[66] Wegelius A., Li X., Turco F., Stensjo K. (2018). Design and characterization of a synthetic minimal promoter for heterocyst-specific expression in filamentous cyanobacteria. PLoS ONE.

[67] Angermayr S.A., Rovira A.G., Hellingwerf K.J. (2015). Metabolic engineering of cyanobacteria for the synthesis of commodity products. Trends Biotechnol..

[68] Dexter J., Armshaw P., Sheahan C., Pembroke J.T. (2015). The state of autotrophic ethanol production in Cyanobacteria. J. Appl. Microbiol..

[69] Wijffels R.H., Kruse O., Hellingwerf K.J. (2013). Potential of industrial biotechnology with cyanobacteria and eukaryotic microalgae. Curr. Opin. Biotechnol..

[70] Anderson S.L., McIntosh L. (1991). Light-activated heterotrophic growth of the cyanobacterium synechocystis sp strain PCC-6803—A blue-light-requiring PROCESS. J. Bacteriol..

[71] Rippka R., Deruelles J., Waterbury J.B., Herdman M., Stanier R.Y. (1979). Generic assignments, strain histories and properties of pure cultures of Cyanobacteria. J. Gen. Microbiol..

[72] Gao Z.X., Zhao H., Li Z.M., Tan X.M., Lu X.F. (2012). Photosynthetic production of ethanol from carbon dioxide in genetically engineered cyanobacteria. Energ. Environ. Sci..

[73] Vidal R. (2017). Alcohol dehydrogenase AdhA plays a role in ethanol tolerance in model cyanobacterium Synechocystis sp PCC 6803. Appl. Microbiol. Biotechnol..

[74] Ehira S., Takeuchi T., Higo A. (2018). Spatial separation of photosynthesis and ethanol production by cell type-specific metabolic engineering of filamentous cyanobacteria. Appl. Microbiol. Biotechnol..

[75] Masukawa H., Mochimaru M., Sakurai H. (2002). Disruption of the uptake hydrogenase gene, but not of the bidirectional hydrogenase gene, leads to enhanced photobiological hydrogen production by the nitrogen-fixing cyanobacterium Anabaena sp PCC 7120. Appl. Microbiol. Biotechnol..

[76] Gartner K., Lechno-Yossef S., Cornish A.J., Wolk C.P., Hegg E.L. (2012). Expression of Shewanella oneidensis MR-1 FeFe -Hydrogenase Genes in Anabaena sp Strain PCC 7120. Appl. Environ. Microbiol..

[77] Khamees H.S., Gallon J.R., Chaplin A.E. (1987). The pattern of acetylene-reduction by cyanobacteria grown under alternating light and darkness. Br. Phycol. J..

[78] Raleiras P., Khanna N., Miranda H., Meszaros L.S., Krassen H., Ho F., Battchikova N., Aro E.M., Magnuson A., Lindblad P. (2016). Turning around the electron flow in an uptake hydrogenase. EPR spectroscopy and in vivo activity of a designed mutant in HupSL from Nostoc punctiforme. Energ. Environ. Sci..

[79] Liang F.Y., Englund E., Lindberg P., Lindblad P. (2018). Engineered cyanobacteria with enhanced growth show increased ethanol production and higher biofuel to biomass ratio. Metab. Eng..

[80] Watt I.N., Montgomery M.G., Runswick M.J., Leslie A.G.W., Walker J.E. (2010). Bioenergetic cost of making an adenosine triphosphate molecule in animal mitochondria. Proc. Natl. Acad. Sci. USA.

